# The middle cerebral artery early branches classification (MCA-EB): a novel imaging-based approach

**DOI:** 10.3389/fneur.2026.1879376

**Published:** 2026-07-08

**Authors:** Arkadiusz Kacała, Małgorzata Milnerowicz-Bescond, Maciej Ziomek, Marek Łukasiewicz, Łukasz Noszczak, Weronika Wrona, Wiktoria Wdowiak, Mateusz Dorochowicz, Kamil Litwinowicz, Anna Zimny, Maciej Guziński

**Affiliations:** 1 Department of General, Interventional and Neuroradiology, Wroclaw Medical University, Wrocław, Poland; 2 Department of Diagnostic and Interventional Neuroradiology, CHU de Bordeaux Pôle d’imagerie médicale, Bordeaux, France; 3 Student research club of General Radiology and Neuroradiology, Faculty of Medicine, Wroclaw Medical University, Wrocław, Poland; 4 Department of General, Interventional and Neuroradiology, Wroclaw University Hospital, Wrocław, Poland

**Keywords:** anatomical variations, classification, computed tomography angiography, early branches, middle cerebral artery (MCA)

## Abstract

**Background:**

The middle cerebral artery (MCA) is one of the most clinically relevant cerebral vessels. Although early branches (EBs) of the MCA have been described, existing classifications are limited by small sample sizes and incomplete representation of anatomic variants.

**Purpose:**

To propose and validate a revised imaging-based classification (MCA-EB) of early branches of the MCA using computed tomographic angiography (CTA).

**Materials and Methods:**

This retrospective study included 200 adult patients who underwent computed tomographic angiography (CTA) between 2020–2024. Patients with intracranial arterial lesions, stenosis, mass effect, or poor contrast opacification were excluded. Morphometric measurements of MCA EBs were performed with dedicated software. Presence, type, and dimensions of early temporal (ETB) and frontal branches (EFB) were assessed. A revised classification system (MCA-EB) was developed based on EB number and distribution. Statistical analyses included Kendall’s tau-b, Spearman correlation, and Cohen’s kappa for reproducibility analysis.

**Results:**

A total of 271 EBs were identified in 215 MCAs (53.25%). ETBs were more frequent (197/271, 72.7%) than EFBs (74/271, 27.3%). Mean diameter was 0.86 mm for ETBs and 0.83 mm for EFBs. No significant correlation was found between EB diameter and distance from MCA origin (ETB1 *τ* = −0.009, *p* = 0.86; EFB1 τ = −0.148, *p* = 0.09). The MCA-EB classification comprised four types with distinct subtypes, ranging from absence of EBs (Type I, 46.3%) to combined ETB and EFB variants (Type IV, 9%).

**Conclusion:**

MCA-EB provides a simplified and standardized classification of MCA early branches. This system may aid in surgical planning, improve recognition of anatomic variants, and support future research on the clinical relevance of MCA EBs.

## 1 Introduction

The middle cerebral artery (MCA) is one of the largest brain vessels that supplies the majority of the lateral surface of the hemisphere, as well as a part of the frontal lobe, and is a frequent location for surgically treatable intracranial aneurysms (1, 2). Various anatomical variations have been associated with the prevalence of neurovascular diseases (2, 3), and although the MCA has been the focus of numerous anatomical studies, only a limited number of reports have examined the characteristics of early branches originating from the prebifurcation trunk of the MCA. Most of the existing studies were carried out on small samples of patients, making the current classification an inadequate reflection of the statistical prevalence of more uncommon early branch types (1, 4).

This study was based on computed tomographic angiography (CTA), and its primary aim was to standardize the anatomical classification and establish a statistically representative, large-scale framework characterizing the early branches of the MCA.

## 2 Methods

Using the RIS database, a total of computed tomographic angiography (CTA) examinations of 200 adult patients were selected, all of whom had no detectable cerebral arterial lesions on CTA scans, and the radiodensity of MCA was measured at at least 250 HU during the examination. The exclusion criteria were: (1) ischemic stroke, or an aneurysm in the MCA, (2) stenosis or blockage in the internal cerebral artery, (3) mass effects (hematoma, hemorrhage, neoplasm), (4) advanced atherosclerosis, post-stroke status, poor contrast opacification in the artery, motion artifacts, (5) agenesis, or dolichoectasia of the MCA and obstruction in the CCA.

Measurement of the morphological data relating to the MCA was performed using AW Server software. For selected patients, specific dimensions were taken from their angiography CT scans and listed in the table. The presence of early frontal (EFB) and early temporal branches (ETB) was reported. Their amount as well as the number of terminal branches was determined. The diameter and the distance of EFBs and ETBs were taken, including both types of distances - from the beginning of the MCA and from the branching of the MCA.

Ultimately, “MCA - EB”, the modified version of the classification system introduced by by Tanriover, et al., (4) was proposed to categorize each of the reviewed early branches (EBs) of the MCA.

### 2.1 Statistical analysis

Statistical analyses were performed using dedicated software. The normality of continuous data, including early branch diameters and anatomical distances, was formally verified using the Shapiro–Wilk test. Because the morphological parameters deviated significantly from a normal distribution (*p* < 0.05), non-parametric correlation methods—specifically Kendall’s tau-b and Spearman’s rank correlation – were utilized to evaluate the relationships between branch diameters and distances from the MCA origin. To evaluate the reproducibility and scientific rigor of the proposed classification system, an intraobserver and interobserver reliability assessment was conducted. A subset of 50 randomly selected CTA examinations was independently reviewed by two senior radiologists. To assess intraobserver reproducibility, the primary investigator re-evaluated the same subset after a 4-week interval. The level of agreement was quantified using Cohen’s kappa statistics. A post-hoc statistical power analysis was also performed to ensure the sample size of 400 M1 segments was sufficiently powered to detect anatomical variations.

## 3 Results

### 3.1 Characterization of MCA’S early branches

Cortical branches originating from the MCA M1 segment are referred to as EBs. Those supplying the temporal area are called early ETBs, while those supplying the frontal area are EFBs. Additionally the first or only originating ETB was referred to as ETB1 and the second as ETB2. Analogous abbreviations were used for EFBs (EFB1 and EFB2).

215 MCAs with at least one EB originating from it and 271 EBs in total were identified. The mean diameter and the distance of EBs from the beginning of the MCA and from the branching of the MCA were summarized in Table 1.

**Table 1:** Characteristics of MCA’s early branches.

Type of EB	No. of EBs (%)	Mean diameter (range) [mm]	The mean distance between MCA origin and EB origin (range) [mm]	The mean distance between EB origin and MCA division (range) [mm]
ETB	197 (72.7)	0.86 (0.3–2.1)	13.24 (4.5–43.8)	10.83 (2.6–44.8)
ETB1	182 (67.2)	0.86 (0.3–2.1)	12.72 (4.5–33.7)	11.04 (2.6–44.8)
ETB2	15 (5.5)	0.84 (0.4–1.6)	19.59 (10.9–43.8)	8.37 (3.8–20.00)
EFB	74 (27.3)	0.83 (0.4–1.8)	15.22 (3.7–36.00)	13.26 (2.1–30.8)
EFB1	69 (25.5)	0.84 (0.4–1.8)	14.89 (3.7–36.00)	13.21 (2.1–30.8)
EFB2	5 (1.8)	0.68 (0.4–1.1)	19.84 (10.7–31)	13.96 (7.4–20.5)

182 MCAs with at least one ETB originating from it were identified. 167 MCAs had one ETB and 15 had two ETBs. The mean ETB diameter was 0.86 mm. The mean distance between MCA origin and ETB origin was 13.24 mm. The mean distance between the ETB origin and MCA division was 10.83 mm.

Similarly, 69 MCAs had EFBs originate from them. 64 had one EFB and 5 had two of them. The mean EFB diameter was 0.83 mm. The mean distance between MCA origin and EFB origin was 15.22 mm. The mean distance between EFB origin and MCA bi−/trifurcation was 13.26 mm. The termination patterns of ETBs and EFBs were summarized in Table 2.

**Table 2:** Frequency of number of EBs terminal branches.

Type of EB	No of EB branches	Frequency	Proc (%)
ETB 1	1	38	20.88
	2	142	78.02
	3	2	1.10
ETB 2	1	7	46.67
	2	8	53.33
EFB 1	1	9	13.04
	2	58	84.06
	3	2	2.90
EFB 2	1	2	40
	2	2	40
	3	3	20

No significant correlations were found between EB diameter and distance from the MCA origin, or between the number of EB terminal branches and their distance from the MCA origin. Regarding reproducibility, the intraobserver analysis demonstrated an excellent reproducibility with a Cohen’s kappa value of = 0.91. The interobserver agreement between the two independent readers was also excellent, yielding a kappa value of 0.86. Post-hoc power analysis confirmed that the sample size of 400 arteries achieved a statistical power of 88% in verifying the lack of distance-to-diameter correlation.

### 3.2 Classification of MCA’S early branches

In this study, the early branches of the MCA were classified according to an anatomical model adapted from Tanriover et al. (4), with modifications to suit our research parameters. In the process, the MCA - EB classification was created. The M1 segment was categorized into four main types, each with specific subtypes based on the origin and combination of early temporal and frontal branches.

Type I: No early branches originating from the M1 segment. This type was observed in 185 arteries.

Type II: Presence of Early Temporal Branches (ETBs) originating from the M1 segment. This type was subdivided into:

- Subtype A: One ETB originating from the M1 segment. Found in 132 arteries.
- Subtype B: Two ETBs originating from the M1 segment. Found in 14 arteries.

Type III: Presence of Early Frontal Branches (EFBs) originating from the M1 segment. This type was subdivided into:

- Subtype A: One EFB originating from the M1 segment. Found in 31 arteries.
- Subtype B: Two EFBs originating from the M1 segment. Found in 2 arteries.

Type IV: Combination of both ETBs and EFBs originating from the M1 segment. This type was subdivided into:

- Subtype A: One ETB and one EFB originating from the M1 segment. Found in 32 arteries.
- Subtype B: Two ETBs and one EFB originating from the M1 segment. Found in 1 artery.
- Subtype C: One ETB and two EFBs originating from the M1 segment. Found in 3 arteries.

**Figure 1:**
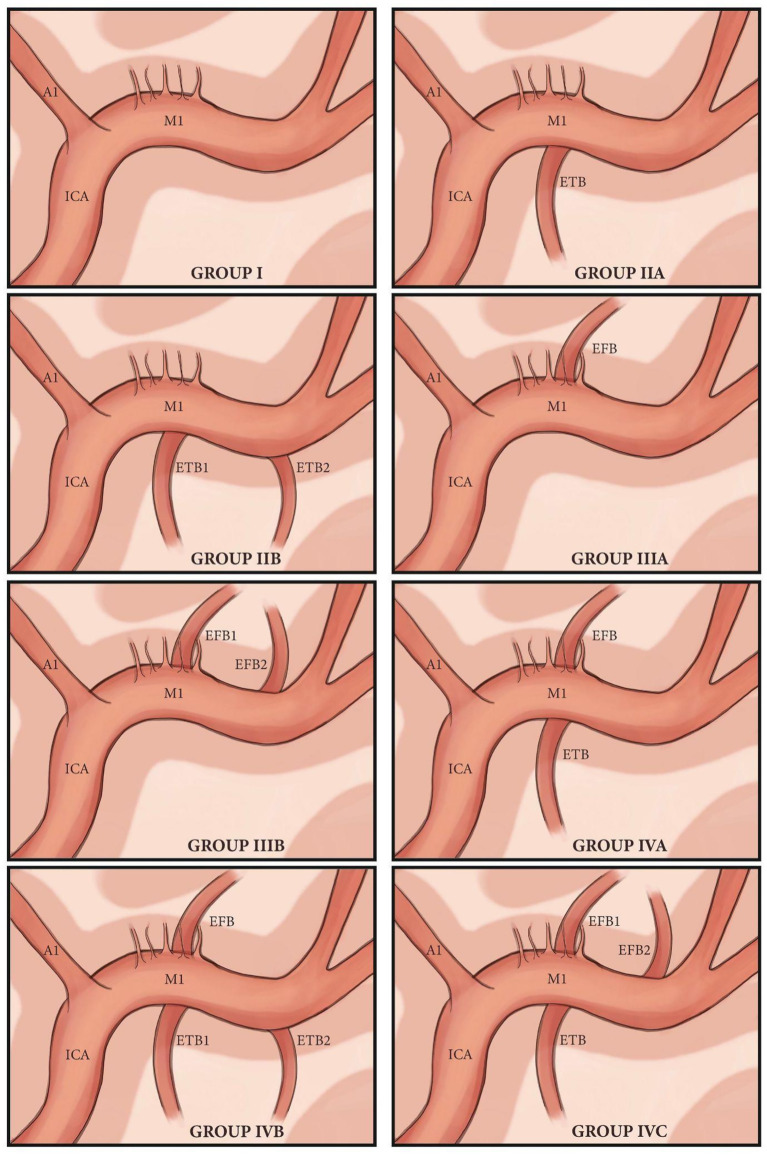
Schematic illustration of the MCA-EB classification, showing the main types and subtypes of early branches of the middle cerebral artery.

**Figure 2:**
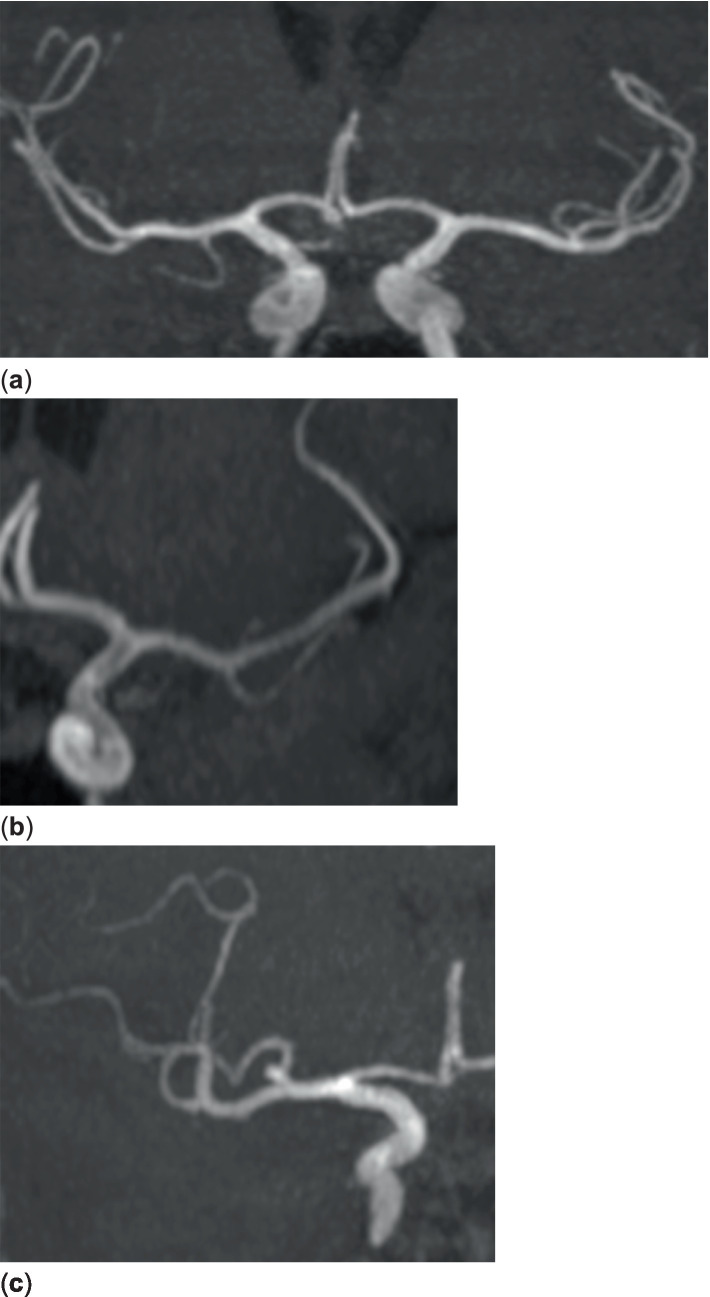
Diagnostic examples of selected MCA early branch variants: **(A)** Right MCA with one ETB (Type IIa) and left MCA with one bifurcated EFB (Type IIIa); **(B)** Left MCA with one ETB and one smaller EFB (Type IVa); **(C)** Right MCA with one EFB and an aneurysm at its origin.

The classification is summarized in Table 3 and illustrated schematically in Figure 1, with additional diagnostic examples of selected MCA early branch variants shown in Figure 2.

**Table 3:** Frequency of M1 segment classification types.

Typ e	Subtype	Description	Number of arteries found	Frequency (%)
I	–	No early branches originating from M1 segment	185	46.25%
II	A	One ETB originating from M1 segment	132	33.00%
	B	Two ETBs originating from M1 segment	14	3.50%
III	A	One EFB originating from M1 segment	31	7.75%
	B	Two EFBs originating from M1 segment	2	0.50%
IV	A	One ETB and one EFB originating from M1 segment	32	8.00%
	B	Two ETBs and one EFB	1	0.25%
	C	One ETB and two EFBs	3	0.75%

## 4 Discussion

The anatomy of the MCA is a critical topic in neurovascular research due to its significant role in cerebral circulation and its association with various clinical conditions (2). Understanding the anatomical variations of the EBs of MCA is essential for both diagnostic and surgical interventions. Despite its importance, research on MCA anatomy, especially the characteristics of early branches, has been limited, with most studies based on small sample sizes.

Few studies have explored the early branches of MCA, and the sample sizes in previous research have been too small to account for most uncommon variants. Early cortical branches may provide collateral blood supply to temporal and frontal lobes and to basal ganglia. Komiyama et al. noticed, that the area supplied by the duplicated MCA (dMCA) matches the area supplied by early temporal branches, and the area supplied by the accessory MCA (accMCA) matches the area supplied by the early frontal branches. Thus, Komiyama et al. (5) suggested that those MCA anomalies might have a common embryologic origin with early cortical branches, expressing early ramification of the MCA. There were reported cases of accMCA providing collateral blood supply in case of main MCA occlusion, sparing at least the anterior part of the frontal lobe (6, 7). We lack reports about dMCA and early branches providing collateral blood supply during ischemic stroke, but we may assume that EFBs can provide similar cortical collateral blood supply to accMCA.

Our research found that approximately 53.25% MCAs exhibited at least one EB. This finding aligns closely with similar studies (2, 8, 9), though other research reports a somewhat higher prevalence of early branching in MCAs (4, 10–12). Our study identified a total of 271 early branches, predominantly ETBs, which aligns with established observations of ETBs being more common than EFBs (4, 8, 11–14). ETBs typically supply the regions of the anterior temporal lobe (e.g., temporopolar or anterior temporal areas) and EFBs supply the regions of the anterior frontal lobe (e.g., orbitofrontal or prefrontal areas). Due to the limitations of CTA compared to microsurgical studies (4, 13, 14), we did not examine the specific cortical areas supplied by early branches.

The origin of early branches, especially for EFBs may mimic the site of MCA division, which results in false bifurcation detection (4, 15). Therefore, some authors emphasize the difficulty in determining the true site of the MCA division and propose their methods to avoid such mistakes (15, 16). Ulm et al. proposed an algorithm: If the main division is recognized at the site of EFB origin, our post-bifurcation inferior branch will send its cortical branches to the frontal lobe, which would not have taken place in the case of a properly recognized division’s site. We investigate the next location of the suspected main division and repeat this process until we find the true division location. Elsharkawy et al. instead of following MCA proximal to distal, proposed following MCA M2 trunks “backwards” to the site of proper division. We believe that a combination of both may help to avoid false bifurcation detection.

In 2000, Türe et al. (12), proposed a classification in which the MCA pre-bifurcation trunk was categorized into types A, B, C and D (TEBC major types II, IV, III and I respectively). 3 years later Tanriover et al. (4) modified Türe’s et al. (12) classification by rearranging it and adding further subtypes.

We proposed MCA-EB, a modified version of the Tanriover Early Branches Classification (TEBC), incorporating additional subtypes based on our findings. We modified TEBC by reducing type II into subtype IIA and IIB, dividing type III into subtypes IIIA and IIIB. The primary novelty of the MCA-EB classification lies in its optimized clinical utility for non-invasive modern imaging and its grounding in rigorous statistical validation. To clarify the precise modifications made to historical frameworks, we provide a direct comparison with previous classification paradigms in Table 4.

**Table 4:** Comparative overview of MCA pre-bifurcation branch classifications.

Feature/criterion	Türe et al. (12) Classification (2000)	Tanriover et al. (4) classification (TEBC) (2003)	Proposed MCA-EB classification (2026)	Rationale for modification/simplification
Primary criteria	Presence or absence of early branches (Types A–D) (12).	Branch presence, specific distal/proximal origin, cortical area size, and number of lenticulostriate perforators (LSAs) (4).	Number and anatomical destination of early branches (Temporal vs. Frontal).	Streamlines clinical image interpretation without requiring micro-dissection parameters.
Distance-to-diameter correlation	Not assessed (12).	Included; assumed that distal branches have smaller diameters (4).	Excluded based on statistical analysis ($*p* > 0.05$) showing no correlation.	Our statistical analysis proved that distinguishing between “terminal” and “distant” branches lacks mathematical justification.
Frontal branch evaluation (EFB)	Not specifically separated from temporal branches (12).	Included, but heavily bundled with combined Type IV variants; isolated EFB variants remained underexplored (4).	Expanded Type III into distinct Subtypes IIIA (one EFB) and IIIB (two EFBs).	Accommodates isolated EFB variants observed in large-scale modern imaging data.
Complexity and clinical utility	Low granularity (anatomical only) (12).	High complexity; difficult to apply completely using routine non-invasive clinical imaging (CTA/MRA) (4).	High universality and scannability optimized for CTA datasets.	Simplifies reporting for interventional neuroradiologists and neurosurgeons during acute setting evaluations.

Furthermore, we proposed the simplification of TEBC. The modification proposed by Tanriover et al. (4) has not solely referred to the presence or absence of early branches as Türe’s classification did but also included their proximal/distal origin in relation to ICA bifurcation, changes in the diameter (ETBs’ diameter decreased the more distal origin it has), size of supplied cortical area and number of given perforators (LSAs). A considerably larger sample size provided a more comprehensive overview of the properties of early branches. A crucial finding was that there is no statistical correlation between the distance of EB from the beginning of the MCA and their diameter. In the widely cited classification by Tanriover et al. (4) (TEBC), the authors incorporated a subdivision based on proximal versus distal origins, postulating that ETB diameters systematically decreased when originating more distally. Our statistical analysis, derived from a considerably larger patient sample, demonstrates that this distinction is not mathematically significant. Consequently, omitting distance-dependent sub-classifications allowed us to introduce a substantially more parsimonious and universal model. Adopting overly intricate historical criteria forces future investigators to classify standard anatomical variations as true anomalies. We believe our findings demonstrate that the simplified classification is more suitable for large-scale research on MCA EBs. This finding led us to conclude that distinguishing EBs into terminal and distant categories is not statistically significant and should not be considered when developing a standardized classification system. This, in turn, allowed us to further simplify the classification. Adopting previously suggested classifications would compel future researchers to treat any EB variant that did not meet all criteria as an anomaly. We believe our findings demonstrate that the simplified classification is more suitable for large-scale research on MCA EBs. Furthermore, since our study did not include analysis of factors such as the size of the supplied cortical area or the number of perforating lenticulostriate arteries (LSAs), we do not believe it contradicts previous research. However, we view these factors as consequences of the anatomy of MCA EBs and propose they not be considered essential components of the classification. The classification proposed in this study aims to be more universal. Therefore we propose that the classification should include the number of EBs and their distinction into ETBs and EFBs. Moreover, EFBs’ samples gathered in past studies were relatively small in size and often occurred alongside ETBs as part of the type IV variant. This may have contributed to incomplete data representation, leaving type III variants unexplored (4, 10). This prompted us to add IIIA and IIIB subtypes. Lee et al. also reported some underdocumented subtypes, mainly concerning major type IV (10). Their study also utilized TEBC and further modified it by rearranging the type IV subtypes. These inconsistencies among researchers highlight the need for future studies to standardize the classification. During the course of our study, we identified and classified several rare subtypes of MCA EBs that had not been previously described in the literature. These findings suggest that the anatomical variability of the MCA EBs is greater than initially understood. Given the inherent complexity and diversity of cerebral vasculature, it is reasonable to hypothesize that additional, yet-undiscovered subtypes of MCA EBs may exist. The absence of these subtypes in our study could be attributed to their extreme rarity. Although the anatomy of MCA EBs remains under-researched, we believe it is a critical topic for understanding neurovascular health and disease. Therefore, the need for a universal classification system, which can be applied in future studies to standardize outcomes, is paramount.

### 4.1 Clinical implications

While this retrospective study evaluates baseline anatomical architecture rather than direct clinical outcomes, several compelling hypotheses regarding neurovascular pathology can be drawn. Structurally, early branches possess small luminal diameters, yet they supply well-defined cortical territories comparable to separate M2 or M3 segments. Consequently, these small calibers indicate that EBs are highly susceptible sites for medium vessel occlusion (MeVO) strokes, rendering them potential candidates for selective MeVO mechanical thrombectomy (17).

Additionally, the origin of early branches is a recognized site for focal hemodynamic stress, creating a potential zone for intracranial aneurysm formation, as demonstrated in our diagnostic series (Figure 2) (15, 16, 18–22). Because lenticulostriate perforating arteries (LSAs) can occasionally emerge directly from early branches—particularly EFBs—the surgical clipping or endovascular coiling of these proximal trunk aneurysms carries an inherent risk of inadvertent branch occlusion, potentially resulting in lacunar infarction in the deep gray matter (15, 16). Some authors already reported such incidents (19–21). Recognizing these specific variations preoperatively through a standardized framework like MCA-EB could assist in optimizing surgical approach selection and refining risk mitigation strategies.

### 4.2 Limitations

Our study has several limitations. Even though our sample size included a bigger sample size, further studies are required to confirm our findings. The low occurrence of some of the subtypes emphasizes the need for larger-scale research on the anatomy of early MCA branches. Only a few studies have focused on describing EBs’ variants and even fewer have concentrated on classifying these findings. Therefore there is a need for more research dedicated to MCA early branches. Furthermore, this study was performed using computed tomographic angiography (CTA). On the one hand, the use of medical imaging tests allowed us to assemble a larger sample size compared to microsurgical studies. On the other hand, CTA possesses lower spatial resolution thresholds than digital subtraction angiography (DSA) or direct microsurgical dissection. This physical threshold may lead to the underdetection of minute, sub-millimeter early branches or tiny, accessory lenticulostriate perforating arteries. Moreover, anatomical studies offer additional information in terms of the supplied specific cortical areas and the presence of LSAs. Furthermore, because we analyzed anonymized imaging datasets of intact vasculatures to establish anatomical norms, we did not evaluate live collateral flow dynamics or real-time ischemic outcomes during acute stroke phases. Future prospective clinical studies are required to validate the translational utility of the MCA-EB classification in active neurosurgical cohorts.

## 5 Conclusion

In summary, the validated MCA-EB classification system simplifies and standardizes the anatomical characterization of middle cerebral artery early branches using an imaging-based approach. By demonstrating an absolute lack of distance-to-diameter correlation, our data justify a streamlined, user-friendly framework centered on branch count and destination. The excellent interobserver and intraobserver reproducibility rates confirm that this system can be reliably applied in radiologic reporting and neurosurgical planning, laying a robust foundation for future prospective trials investigating MeVO stroke management and proximal aneurysm treatments.

## Data Availability

The raw data supporting the conclusions of this article will be made available by the authors, without undue reservation.

## References

[1] De Long WB. Anatomy of the middle cerebral artery: the temporal branches. Stroke. (1973) 4:412–8. doi: 10.1161/01.STR.4.3.412, 4713030

[2] Oo EM Saw KEE Oo HN Than T Thida K. Variable anatomy of the middle cerebral artery from its origin to the edge of the Sylvian fissure: a direct fresh brain study. ScientificWorldJournal. (2021) 2021:6652676. doi: 10.1155/2021/6652676 33776597 PMC7969099

[3] Caranci F Briganti F Cirillo L Leonardi M Muto M. Epidemiology and genetics of intracranial aneurysms. Eur J Radiol. (2013) 82:1598–605. doi: 10.1016/j.ejrad.2012.12.026, 23399038

[4] Tanriover N Kawashima M Rhoton AL Ulm AJ Mericle RA. Microsurgical anatomy of the early branches of the middle cerebral artery: morphometric analysis and classification with angiographic correlation. J Neurosurg. (2003) 98:1277–90. doi: 10.3171/jns.2003.98.6.1277, 12816276

[5] Komiyama M Nakajima H Nishikawa M Yasui T. Middle cerebral artery variations: duplicated and accessory arteries. AJNR Am J Neuroradiol. (1998) 19:45–9. 9432156 PMC8337320

[6] Komiyama M Nishikawa M Yasui T. The accessory middle cerebral artery as a collateral blood supply. AJNR Am J Neuroradiol. (1997) 18:587–90. 9090429 PMC8338391

[7] Liu Z-S Zhou L-J Sun Y Kuang X-W Wang W Li C. Sufficient collateral blood supply from accessory middle cerebral artery in a patient with acute ischemic stroke. Interv Neuroradiol. (2015) 21:215–7. doi: 10.1177/1591019915583230, 25943843 PMC4757236

[8] Ogeng’o JA Njongo W Hemed E Obimbo MM Gimongo J. Branching pattern of middle cerebral artery in an African population. Clin Anat. (2011) 24:692–8. doi: 10.1002/ca.21147, 21374730

[9] Vuillier F Medeiros E Moulin T Cattin F Bonneville J-F Tatu L. Main anatomical features of the M1 segment of the middle cerebral artery: a 3D time-of-flight magnetic resonance angiography at 3 T study. Surg Radiol Anat. (2008) 30:509–14. doi: 10.1007/s00276-008-0360-3, 18465079

[10] Lee CS Park JC Park J-K Sim K-B. Angiographic pattern in the early branches of the middle cerebral artery. J. Korean Soc Radiol. (2011) 64:103. doi: 10.3348/jksr.2011.64.2.103

[11] Idowu OE Shokunbi MT Malomo AO Ogunbiyi JO. Size, course, distribution and anomalies of the middle cerebral artery in adult Nigerians. East Afr Med J. (2002) 79:217–20. doi: 10.4314/eamj.v79i4.8883, 12625681

[12] Türe U Yaşargil MG Al-Mefty O Yaşargil DC. Arteries of the insula. J Neurosurg. (2000) 92:676–87. doi: 10.3171/jns.2000.92.4.0676, 10761659

[13] Gibo H Carver CC Rhoton AL Lenkey C Mitchell RJ. Microsurgical anatomy of the middle cerebral artery. J Neurosurg. (1981) 54:151–69. doi: 10.3171/jns.1981.54.2.0151, 7452329

[14] Umansky F Juarez SM Dujovny M Ausman JI Diaz FG Gomes F . Microsurgical anatomy of the proximal segments of the middle cerebral artery. J Neurosurg. (1984) 61:458–67. doi: 10.3171/jns.1984.61.3.0458, 6747682

[15] Ulm AJ Fautheree GL Tanriover N Russo A Albanese E Rhoton AL . Microsurgical and angiographic anatomy of middle cerebral artery aneurysms: prevalence and significance of early branch aneurysms. Neurosurgery. (2008) 62:ONS344–53. doi: 10.1227/01.neu.0000326018.22434.ed, 18596514

[16] Elsharkawy A Lehečka M Niemelä M Billon-Grand R Lehto H Kivisaari R . A new, more accurate classification of middle cerebral artery aneurysms: computed tomography angiographic study of 1,009 consecutive cases with 1,309 middle cerebral artery aneurysms. Neurosurgery. (2013) 73:94–102. doi: 10.1227/01.neu.0000429842.61213.d5, 23615110

[17] Ospel JM Goyal M. A review of endovascular treatment for medium vessel occlusion stroke. J Neurointerv Surg. (2021) 13:623–30. doi: 10.1136/neurintsurg-2021-017321, 33637570

[18] Brzegowy P Polak J Wnuk J Łasocha B Walocha J Popiela TJ. Middle cerebral artery anatomical variations and aneurysms: a retrospective study based on computed tomography angiography findings. Folia Morphol. (2018) 77:434–40. doi: 10.5603/FM.a2017.0112, 29235088

[19] Park JC Shim JH Lee DH Ahn JS Lee D-G Yang K . Three-dimensional angiographic evaluation of middle cerebral artery trunk aneurysms: demonstration of the close relationship between the early frontal cortical branches and lateral Lenticulostriate arteries. World Neurosurg. (2016) 91:383–9. doi: 10.1016/j.wneu.2016.04.065, 27132178

[20] Abe T Ohnishi M Katsumata A Nishio S Kawauchi M Matsumoto Y. Aneurysms at the early branch of middle cerebral artery. No Shinkei Geka. (2006) 34:383–8. 16613219

[21] Park D-H Kang S-H Lee J-B Lim D-J Kwon T-H Chung Y-G . Angiographic features, surgical management and outcomes of proximal middle cerebral artery aneurysms. Clin Neurol Neurosurg. (2008) 110:544–51. doi: 10.1016/j.clineuro.2008.02.014, 18367320

[22] Matsukawa H Kamiyama H Miyazaki T Kinoshita Y Ota N Noda K . Surgical treatment of middle cerebral artery aneurysms: aneurysm location and size ratio as risk factors for neurologic worsening and ischemic complications. World Neurosurg. (2018) 117:e563–70. doi: 10.1016/j.wneu.2018.06.077, 29929026

